# Correcting the Brain? The Convergence of Neuroscience, Neurotechnology, Psychiatry, and Artificial Intelligence

**DOI:** 10.1007/s11948-020-00240-2

**Published:** 2020-07-06

**Authors:** Stephen Rainey, Yasemin J. Erden

**Affiliations:** 1 Oxford Uehiro Centre for Practical Ethics, Suite 8, Littlegate House, St Ebbes Street, Oxford, OX1 1PT UK; 2 St. Mary’s University, Twickenham, UK

**Keywords:** Neurotechnology, Artificial intelligence, Ethics, Normativity, Psychology, Psychiatry

## Abstract

The incorporation of neural-based technologies into psychiatry offers novel means to use neural data in patient assessment and clinical diagnosis. However, an over-optimistic technologisation of neuroscientifically-informed psychiatry risks the conflation of technological and psychological norms. Neurotechnologies promise fast, efficient, broad psychiatric insights not readily available through conventional observation of patients. Recording and processing brain signals provides information from ‘beneath the skull’ that can be interpreted as an account of neural processing and that can provide a basis to evaluate general behaviour and functioning. But it ought not to be forgotten that the use of such technologies is part of a human practice of neuroscience informed psychiatry. This paper notes some challenges in the integration of neural technologies into psychiatry and suggests vigilance particularly in respect to normative challenges. In this way, psychiatry can avoid a drift toward reductive technological approaches, while nonetheless benefitting from promising advances in neuroscience and technology.

## Introduction

Neurosciences and psychiatry overlap when the identification of anomalous neural activity is mapped to behavioural or cognitive phenomena in the context of assessment or diagnosis of patients. This means in practice that technologies developed for recording neural activity can come to play a role in psychiatry. Given this, there is a clear need to examine not only the relationship between neuroscience and psychiatry, but also the use of neurotechnology in psychiatry. The specifics of how such technologies operate become particularly salient when they are placed in the context of a practice aimed at evaluating human behaviour, such as psychiatry.

In some cases, neurotechnology can rely on artificial intelligence (AI), especially in the prediction, or analysis of neural recording data (Glaser et al. 2017; Kellmeyer 2018). This represents a significant element worth its own investigation, again because it is deployed in a context of evaluating human behaviour. How AI develops and is used in this kind of context is in need of analysis. In this paper the analysis will involve identification of key normative differences between brain-based intelligence and artificial intelligence. To do this we point to some general complexities of human intelligence (HI), especially as based in complex, reasoned activity.

As a means of blending technological advances with human understanding, we recommend discussion that draws upon a variety of discussants and sources of information. This fits with more familiar psychiatric methods involving doctor/patient encounters, which are typically framed as a type of discussion. Even with a power or authority imbalance between psychiatrist and patient, the conversational form of interaction forms the basis on which patients reveal their felt experience for expert appraisal by the psychiatrist. The interpolation of technological norms into these otherwise interpersonal spaces may serve to undermine that space. Technology appears to offer objective answers to problems and so can seem to overshadow the subtleties of more discursive approaches to human problems. Particular care ought to be taken in developing neuropsychiatric accounts of human cognition and behaviour where diagnosis of psychiatric disorder is at stake. This and related issues are central in this paper.

## Neuroscience and Neurotechnology

Neuroscience, in general, promises a growing understanding of how the brain works. As every conscious state is understood as being instantiated by some neural activity, greater understanding of conscious states ought to arise from greater understanding of the brain. Some influential voices have already described psychiatry itself, for instance, as ‘clinically applied neuroscience’ (Insel and Quirion 2005). With accelerating progress in the detection, recording, identification, and decoding of brain signals, technologies play a crucial role in making the brain legible to researchers (Farahany 2018; Haselager and Mecacci 2018; Rose 2016a, b). Nevertheless, how this understanding is applied is significant.

Neural activity is highly organised, and the brain is richly structured with tremendous complexity (Churchland 1989). Given this complexity, it might be easy to conflate a problem manifested in a conscious state—a psychiatric disorder for example—as a result of misfiring neurons, damaged circuits, or poorly connected regions. Nikolas Rose and Joelle Abi-Rached have referred to this kind of scenario as a ‘screen and intervene’ paradigm (Rose and Abi-Rached 2014). In such a state of affairs, the use of technology in detecting neural activity is taken as sufficient for identifying *anomalous* activity upon which interventions are thereby justified. These might be pharmacological, neurotechnological, or even consist in corrective writing to brains (Roelfsema et al. 2018). ‘Writing to brains’, for instance, allows for the modification of neural activity through the creation of electromagnetic fields. These fields are intended to excite, or inhibit neural activity in ways aimed at promoting cognitive and behavioural effects.

In developing a science of the brain, there is a risk of generalising about detectable neural activity from which may emerge hastily constructed accounts of behavioural or cognitive phenomena in terms of this activity. This is connected with the question of how to avoid reducing accounts of human cognition to neuroscientific norms, while at the same time acknowledging the powerful role of neuroscience in addressing human psychiatric disorders. Such reduction could blur the boundaries between organic and functional disorders, hitherto essential to discourse on mental illness, for instance. This blurring also affects the boundary between psychological traits (personality) and psychiatric states (illness). The potential for this reduction of human beings to ‘neurochemical selves,’ rather than persons, ought to be carefully scrutinised (Rose 2003).

The above noted issues are pertinent in evidence-gathering as part of psychiatric assessment or diagnosis. Privileging causal explanations of action, or neurobiologically reductive bases for action and behaviour, may well lead to a sort of *reason*-*curtailment* wherein the scope of reasons available to account for action and behaviour is reduced. This could result in a *too reductive* account of complex human behaviour both in terms of rationality, and action, especially as it relates to the perceptions both of patients and of practitioners. Where behaviours appear to be explained by some causal story supported by data, discursive accounts of those same behaviours may become less influential. This may sound somewhat abstract, but with reference to neurotechnologies, we can offer a useful example of this reductive potential.

## Neurotechnologies, AI and Modifying Neural Activity

Neurotechnologies typically record and decode brain signals for a variety of purposes. These include controlling software and hardware, and providing information for neural monitoring, as in neurofeedback devices (Sitaram et al. 2017). These last-mentioned devices sometimes raise claims to neural or human enhancement. These are ‘open loop’ systems in that they detect, record, and process neural signals happening already, in their own right. The open loop neurotechnological device is akin to a spectator, its outputs simply repeating what is happening under the skull, instrumentalised somehow. In such instances, AI may be a part of the system overall, especially used in the prediction, visualisation, or analysis of neural recording data.

Other instances of neurotechnology include neural stimulation as part of their function. For instance, deep brain stimulation (DBS) stimulates neural regions and in doing so modifies neural activity. Typically, this is done in order to diminish deleterious symptoms of diseases like the shaking seen in Parkinson’s disease (Fang and Tolleson 2017). The strength and the duration of these kinds of DBS interventions can be monitored and altered by the user, and the devices can also be turned on and off. In terms of AI, this specific Parkinson’s application of stimulation technology appears not to be highly problematic. This application is often user-controlled. But especially in applications that are not approved treatments, as DBS is for Parkinson’s, the convergence of AI and neural stimulation can raise other issues.

A closed-loop neurotechnological device is capable of detecting, and modifying neural activity. This would allow for detailed creation of a desired neural state, and its maintenance via stimulation. Given the complexity of this task, software control systems would be required to operate the maintenance function. The creation and maintenance of a given neural state would require close attention to the state of the neural region to be monitored, as well as real-time intervention to keep it on track with the desired state (Xu et al. 2014). Unlike systems such as DBS, this kind of control would be too fine-grained for a person to manage. For these reasons, AI enhanced technology may be deployed to a greater extent than simply the recording or analysis of neural data. AI technologies with software-controlled closed-loop neurotechnology raise questions concerning free will, agency, responsibility, and perception. For instance, it is not clear what control a patient might have over the technology, or what impact might be felt if the technology fails. At the very least, and most pertinently here, we know that physical intervention in the brain’s chemical and electrical activity has effects on mental states.

The instrumental potential of neural states also underwrites pharmacological intervention in cognitive and mental states, as well as treatments such as DBS. This is the rationale for using DBS in cases of obsessive compulsive disorder, persistent depression, or anorexia nervosa for instance (Klein et al. 2016; Maslen et al. 2015; Widge and Sahay 2016). It is also key in the development of neurotechnologies, such as neuroprosthetics for speech, which might be seen as likely successors to a neuropharmacological-psychiatric industry (Parastarfeizabadi and Kouzani 2017). At any rate, these instrumentalisations are embedded within a recognisably discursive practise. Psychiatric assessments, despite or because of power imbalances between practitioner and patient, allow for a therapeutic identification of problems wherein psychological traits shade into indicators of disease, as with brain lesions. The regime here is one of observation and discussion, then to report, intervene and treat. The process can be repeated until a satisfactory outcome arises. In the case of a closed-loop neurotechnology controlled by software, this regime is altered.

All functions of observation, intervention, and treatment may be given over to a closed loop neurotechnology system, especially the algorithms governing its ‘good’ functioning (Widge and Sahay 2016). In one sense, the automaticity of the system assumes a 1:1 relation between neural processes and desired mental state, itself the basis for a desirable behavioural outcome. Put another way, the closed-loop neurotechnology setup is predicated on the idea that the creation and maintenance of a given neural state will promote or determine a macro-scale behavioural effect. This may be via mediating that state, or through inhibiting a non-desirable state. What is key here is the power given to the algorithms that assess and modify neural activity, mental states, and thereby perception and action.

Returning to the idea that neural states are closely related to mental states, we can see that closed-loop neurotechnologies present an unusual inversion of familiar empirical ‘experience.’ Conscious states are characterisable in terms of neural signals typical of those states; verbal thinking, problem solving, and emotion, among other activities. While neural states correlate with mental, cognitive, and behavioural states, we do not simply undergo them. Neural activity responds to conscious activity as well as producing it. We *use* our brains in a manner not easily describable in mechanistic terms (Kirmayer and Gold 2011). This points to issues in determinism that are beyond the scope of this paper, but for now it need only be observed that there is at least some sense in which neural states can be made through conscious activity, as much as they are passively undergone. Moreover, all such mental states and neural processes take place within a rich cultural, social, and embodied identity, which codify a set of experiences that an AI-enabled neurotechnology may struggle to encode.

Closed-loop neurotechnology inverts the order as experienced by the user and instead prioritises a top-down account of brain-consciousness-activity to create a mental state via neurointervention. Unlike DBS treatment for Parkinson’s, there is no ‘human in the loop’ on this neurotechnology model. This means that AI-enabled software manages neural state monitoring as well as maintenance. By hypothesis, this limits the scope for the closed-loop neurotechnology user to change her mind, for instance (Glannon 2016). Where a user cannot easily intervene to modify or switch off a system, this appears to offer a direct challenge to will and agency (Goering et al. 2017), as well as broader conceptual problems for the conflation of technological and psychological norms.

For instance, if we go for a drive in a car, we can always turn back if we decide that the initial decision was a bad one, or if conditions change and make the drive undesirable. Can we say the same for the closed loop neurotechnology user concerning their own mental states? The initial decision to use the system, the initial ‘pro-neurotechnology’ stance, may be seen as triggering a chain of events sealed off from revision in the light of changed minds or negative externalities. The nature of the closed loop neurotechnology may exacerbate this ‘sealing off’ owing to its creation and maintenance of *neural states* designed to promote or preclude given *mental states*. These states, more to the point, are decided upon prior to the present, in the initial calibration of the neurotechnology system. The conditions hit upon at that point in time are then reinforced algorithmically at every point along the line. The user’s present neural states are thus closely predicated on decisions made prior to the present in an intentional, yet rather artificial and unfamiliar way.

As long as a present neural state, predicated on a past state, is created and maintained by algorithm-controlled electrical intervention, the mental states promoted or inhibited by that neural state will presumably curtail full user control of the present state (Tamburrini 2009). This serves to illustrate an issue in responsibility, as the neurotechnology user may determine for themselves a condition that foreshortens their responsiveness to reasons. In curtailing this dimension of themselves, their basis for action appears to be somewhat diminished. This could even amount to a kind of personality change (Gilbert et al. 2017; Klaming and Haselager 2013; Pugh et al. 2018; Temel et al. 2006). Because a neural state is being created and maintained, certain neural signal profiles will be promoted and others diminished. Any mental states associated with, or precluded by, those signal profiles will thus be promoted or diminished. This seems to challenge how we might think conventionally of ‘agency,’ as a kind of hybrid control at play.

Closed-loop neurotechnologies provoke a rational-responsibility problem. In other words, we face a situation whereby the rationale for an action, including as a grounding for responsibility, may be assigned to either an AI-enabled neurotechnology, or to the human actor (see also Rainey 2018). Whereas ‘action’ is typically connected to reasoning, which includes a full gamut of reasons, with neurotechnology we are left with the possibility that reasons may not be available, transparent, or even coherent. In addition, we know that different neural states can underwrite different kinds of perceptual judgements, as seen in transcranial magnetic stimulation (TMS) experiments (Silvanto et al. 2008). So, reasoning is not the only factor likely to be affected by closed-loop neurotechnology; perception is also at stake. In a scenario where action—as the outcome of reasoning—may be curtailed, and basic behavioural discrimination among stimuli is affected, we suggest that great care is taken in the use of these technologies, particularly as pertains to their use in psychiatry.

## Data, Black Boxes, and Reasoning: Making Sense of Brains and Minds

A central part of what is at issue here is the role of AI as decision-maker in the examples above. A kind of hybridised control appears in these cases, the agent herself having more or less limited control. Where the control in question concerns neural states, and thereby mental states, this is all the more acute. AI-powered methods can provide effective predictions about brain activity quickly, and unexpectedly, from huge amounts of data (Bzdok and Meyer-Lindenberg 2018). These methods are often very complex and opaque, and consequently are often not understood well. This is especially the case where there is some ambition to expand the use of machine learning and related systems in psychiatry (Dwyer et al. 2018). While our focus here are the systems used, we are also signalling that the field into which such systems will appear may require further analysis. This includes methodological ramifications which might be very wide-ranging indeed (Kitchin 2014).

Machine learning techniques designed to generalise from complex and varied data in order to predict particular cases tend to rely upon statistical methods. These may be of various types, but a common feature is that they are often deployed effectively as a black box (Samek et al. 2017). This, in general, can be seen as a problem with machine learning approaches. Despite their often impressive successes, these machine learning applications remain inexplicable in some important respects, owing to their mathematical complexity and opaque processing methods. This inexplicability may even be prevalent among those involved with developing the applications (Hart and Wyatt 1990).

In psychiatric contexts, or health more widely perhaps, this black box problem cannot be overlooked. With a typical clinical encounter being akin to a discussion of some kind (power imbalances notwithstanding) a black box technology in that encounter raises ethical as well as conceptual issues. This is all the more pressing, perhaps, if that black box is given some authority in providing evidence in assessing or diagnosing a disorder. This might be said to occur given the role of neural imaging in diagnosis, for instance. In using technologies to identify neurotypical states, and thereby indicating neurodivergence, there is a risk of ‘technological paternalism’ (Hofmann 2003), or untenable faith in the objectivity of a device’s outputs. This would be an unwarranted reduction of self-reported experience to objectively observable neural activity. Similarly problematic is an attempt to go from a neural account of experience to a phenomenological one (Gallagher 2005).

Even with efforts to restructure the patient-clinician relation, the complexity of shared decision-making practices and the possibility for dialogue is not easy to resolve (Thompson 2007). Paternalism in general is less and less popular in modern medical models of patient engagement, as it does not sufficiently prioritise patient autonomy. Yet if this paternalism is displaced into a machine, one that cannot be understood by the patient (and perhaps not even the physician, at least in terms of its processing), additional issues arise. Even if a diagnosis were to be correct, it would have limited legitimacy. This is because a diagnosis may falter when the strategy relies somehow on a process with at least one inexplicable element.

Machine learning approaches might fare better if disease did not include fuzzy concepts, and was not classified in terms of symptoms or descriptions of function (Dwyer et al. 2018). Such factors are interpretable and so prompt variety rather than specificity. Biomarkers, objective criteria, would likely provide a better basis for machine learning based psychiatry. It ought to open the difficult question: is the technology ‘operating better’ equivalent to *psychiatry* operating better?

One issue turns on how diagnosis works in psychiatry, where psychological traits may be scrutinised as psychiatric disorders. The construction of an ideal type of brain function, say, as derived from a mass of tokens examined in research, is instructive for various research aims. But this ought not to serve as a *telos*, or an end toward which any deviation ought to be steered. The ‘datafied brain’ is not the *proper brain* by dint of being based in many instances, and individual deviations from neurotypical models ought not to be thought of as deviant brains per se. This is especially important where neuroscience would hope to inform psychiatry, and where abnormal neural data may be taken to evidence psychiatric disturbance.

In terms of data diversity there is a serious underrepresentation of any but those of Western European origin, signalling the curated nature of data sets (Chiao and Cheon 2016; Gitelman 2013; Henrich et al. 2010). Yet technologies, such as those processing data curated from sub-sets of large groups, appear in some sense to present neurotypical states as objectively proper neural states. As various discussions have served to illustrate, imaging in particular can be problematic in encouraging faulty reasoning (Logothetis et al. 2001; Poldrack 2006). As Joseph Dumit notes, brain scans of patients diagnosed with schizophrenia are referred to by researchers as ‘schizophrenia,’ whereas others are labelled ‘normal controls.’ This reveals a way in which researchers can come to see positive scans as showing ‘schizophrenia itself’ somehow, rather than indicating symptoms. In this example, “…the symptom has been collapsed into the referent” (Dumit 2016, p. 222).

Just as we do not need to pit conventional human intelligence (HI) and AI in opposition, we also do not need to set neuroscience and psychiatry in opposition. As Simon Cohn writes of psychiatry,

The issue is not that neuroscience does not, or should not, play a significant role in psychiatry, but that at present at least its commitment to a simple reductionist paradigm is also affording the researchers a degree of naivety and lack of social awareness that is of concern. The effect is that, unlike traditional psychiatric encounters which, despite issues of power and inequality, are nevertheless inherently social interactions, the emerging role of neuroscience in psychiatry suggests the role of individual experts and doctors might be deferred by the apparently objective, and self-determining technology. (Cohn 2016, 180)

The mind is an ‘open system’ not well accounted for by wholesale reduction (Rose 2016a, 63), even though the physical basis for many of its operations can be well understood through neuroscientific research. This reductionism is similarly unsuccessful when narrow AI accounts of reason-led action are presented as somehow indicative of an idealised HI. Diagnosing problems of the mind in terms of neurophysical anomaly omits key details about what mindedness consists in. In relying too much on black box technologies in the processes of assessment and diagnosis, it is important not to miss this point. For these reasons, the place of AI in these processes requires some further scrutiny.

## Action, Reason, and Norms: Comparing HI and AI

For our purposes here, we propose that AI can include anything that seeks to reproduce or simulate methods of decision-making, and reasoning, by technological means. By HI, we point very broadly to the human ability to *reason*, such that a basis for distinguishing between causal or caused activity and intentional action can be established. On this account, action can be interpreted as being done for reasons, whereas caused activity occurs owing to physical laws.

AI offers powerful and useful tools for neuropsychiatry. AI can be deployed in various contexts, especially in areas of neuroscience where it can outperform human beings in key tasks. Some think that it will have a revolutionary effect across this variety of contexts (Grace et al. 2017), especially for pattern-recognition. It is also thought that AI will have an important role in future clinical research and medical imaging, for instance (Ramesh et al. 2004). AI systems can be used to predict events, especially given the scope for analysing datasets much larger than would be feasible for any given human. Alongside these successes are more common feats including beating humans in games, and sometimes with impressive displays of original moves (Silver et al. 2016). As noted above, AI offers scope for neurotechnologies in the diagnosis and specification of treatments of brain and psychiatric disorders. This next section explores what might be the conceptual limitations of these technologies, and what might be the likelihood that such limitations can be overcome.

Especially in terms of psychiatry, in human reasoning there is a vested interest in examining beliefs, weighing desires, and fitting them with various intentions. From these sorts of activities, HI looks for good reasons for thinking or behaving in different ways. Our starting point here is that AI does not ‘look for reasons,’ let alone look for good ones. While AI may have the appearance of ‘looking for reasons’, in fact it operates according to statistical methods. Unlike human reasoning, beliefs are not examined, desires are not weighed, and intentions are absent. Yet AI has no interests beyond the completion of these actions, and so nothing in this process can be said to be better *for the AI itself* than anything else from an imagined AI point of view. We pass over the question of whether AI could exhibit something like irrationality or mental illness, though perhaps reflection on this idea could aid in understanding HI and the potential disorders of humans in general (Ashrafian 2017).

Understanding HI reason-selection is complex and includes trying to understand the actions of another even when they exhibit odd behaviours. One example is of apparent delusion, and the efforts exerted to engage with such action as meaningful, despite possible delusion. In such circumstances we can and do ask for (and typically expect to receive) some account of why one course of action was chosen over another. For AI, this process is not so simple, despite ongoing work to try to re-create the kinds of justificatory argumentation ubiquitous in HI (Bench-Capon and Dunne 2007). HI and behaviour is rich in reason-giving and in reason-expecting, all of which is essential to our *form of life* (Wittgenstein 2009), to our mental states, as well as to our ethical and legal systems and structures.

We could envisage in principle an AI in the sense of a highly complex simulation of decision-making, modelled on patterns from past cases. But this cannot replicate the complexity of human norms as they appear standardly in much reasoning. AI and machine learning approaches typically rely on statistical analysis of patterns of reasoning. Even when made more complex by fusing different modelling techniques and seeking meta-models by technical means (Dwyer et al. 2018), the norms upon which prediction will proceed are descriptive. They are not acted upon so as to ground ‘good reasons,’ but are simply attractors in a possibility space.

Put simply, when it comes to reasoning in general, the norms we are interested in are those of HI. In specific cases, we can deploy AI to get tasks done efficiently. But in any case where an AI could be deployed to get some task done efficiently, even if that AI really would get the job done, we have the HI-type question of whether that deployment would be a good idea. The answer to that question would draw upon a potentially wide set of evaluable reasons, linked to practical conceptions of oneself and the world at large. In other words, there is typically more at stake in the diagnosis of a disorder than just the correct identification of a disorder. The need to take into account a complex set of cultural and conceptual norms (within which any diagnosis is meaningful) requires highly sophisticated judgements, including that of the patient, of the psychiatrist, and of assorted other actors in the relevant medical and social structures. This includes taking into account whether a diagnosis is useful, for instance.

An essential contrast between AI and HI in these examples is the role of norms. AI systems often run on the basis of descriptive norms, norms derived from statistical analysis of many cases. AI learning that is essentially statistical is thereby essentially inductive. HI can ‘run’ on a variety of norms, including but not limited to the descriptive. Our suggestion is that an AI-based analysis of mental states will not be able to recover non-descriptive norms, and therefore will not be able to recover the whole variety of reasons for action that HI might be using. AI-driven analysis of data relating to humans is necessarily highly focussed, task-driven, and exclusionary. This means that it may not capture the same kind of normative complexity available to human reasoning and judgement. Decontextualised data cannot provide the same content and contexts of the humans that such data are taken to represent.

HI is constrained by ‘rational norms’ which can be grounded in a variety of ways including semantics, politics, and socio-cultural conditions. Such variables are not arbitrary, but they are contingent. They are on the one hand expected and predictable, while also being flexible and surprising. This variety of norms provides a structure for behaviour, and for coordinating behaviour among groups, but it is not a singularly logical structure. While logical structures ground basic sets of norms for both AI and HI, in the case of HI, logical consistency and the validity of arguments isn’t always central to either intelligence or to behaviour, and to assume it will be is to miss a great deal about human rationality. Our reasoning is impacted by the content of our experiences (arguably more than by structure) and our expectations are guided accordingly.

Human beings, as rational beings, are not just bound to look for reasons to act, where any reason will do; we also look for specific, compelling, convincing reasons. In other words, *good* reasons. Because human beings can typically reflect upon varieties of reasons to act in one way or another, or to refrain to act at all, there are evaluative conditions upon reasoning. It is not simply the case that by perceiving a reason to do x as a reason to do x we are rationally bound to do x. Rather, when a reason to do x presents itself, it is as if this reason enters a ‘gap’ between the reason seen as a reason, and the eventual acting upon that reason. This evaluative gap is where HI assesses reasons as good or not, appropriate or not, worthy or not, and so on.

Good reasons are tied to a practical, contextualised, idea of oneself. If such a context were not important, we would just act on whatever reasons came to mind by virtue of their being identifiable as reasons. For example, to say of an unidentified mechanism (with uncertain consequences) that a lever ‘has a handle that fits my hand comfortably, therefore I will pull the handle,’ offers a rather weak and unlikely indicator for either decision-making or action. A less surprising and more relatable type of reasoning here might be, ‘I don’t know what the handle does, so no matter how comfortable it appears, I won’t pull it until I know more.’ If I thought of myself as a discrete series of properties, rather than as a unified whole, and of the world as only a set of circumstances here and now, I might decide to pull the handle. Without a practical conception of myself related to a wider external reality, as Christine Korsgaard puts it, I would lose any sense of having reasons for doing one thing rather than another (Korsgaard 2012).

This is what is meant by ‘looking for good reasons.’ The self, on this account is contingent on my being a reasoning, reason-seeking, reason-bounded creature. This contrasts with cases of AI learning from statistical analysis of data from past examples. An AI that learns by statistical analysis can derive only descriptive reasons to act, based in past cases. These are not necessarily good reasons. Indeed, their status as ‘reasons’ is only available to the interpreter of the data, not the AI itself. The AI is as likely to pull the handle as not.

Our argument here involves the claim that to reduce human reasons to simple gap-filling causes of behaviour is to miss details informed by a rich phenomenological experience of rationality and behaviour. On the one hand, neuroscience has a transformative role in understanding the brain, and a great deal to say in coming to understand mental processes. On the other, this role ought not to be one of determining norms for the mental as derived from the neural. Norms for mental activity, as well as for reasoning and rationality are heterogeneous and heterodox: discussion of them ought to be just as heterogeneous and heterodox. This is particularly important where AI is employed to supplement human reasoning, especially in technologized practices of psychiatry. At the very least, AI may be present in the construction of evidence for neurotypical brains, or desirable neural states, as they relate to ordered and disordered cognition or behaviour. These kinds of evidence may supplement psychiatrists’ reasoning by providing clinical decision support. Strange behaviour, coupled with neurodivergence, may be seen as a strong basis for a specific kind of diagnosis, for example.

The normative point is that the neurotypical, in this example, is not constructed by AI techniques *as an ideal*, but is simply as the outcome of a complex but algorithmic procedure. Its use in diagnosis as an ideal, divergence from which is indicative of problems, is too blunt a tool. In a highly discursive psychiatric practice, explanations are forthcoming for behaviour from a variety of sources, not limited to comparisons with neurotypical exemplar states. The point here is to warn against a drift toward relying too much on the apparent objective normativity of evidence gained from sources like AI-derived neurotypical brains. This brings us back to reasons.

## Interpreting Strange Reasons

The general expectation is that human beings act on reasons, and that when they do not obviously, clearly, or consistently act on reasons, then a reason may still be offered for such divergence. This is the case when, for instance, a person can be said to have temporarily ‘lost their mind,’ behaved otherwise than might be expected, or when a person is described as being victim to their impulses, moods, or to some other emotion or impairment. It is also at least part of what may be meant when we talk of someone having a mental health issue.

The expectations displayed in this terminology, and indeed the expectations of doctors and associated experts and carers, are that there is a person who is or has the potential to be rational. From this first, basic expectation of a person as capable of reason, we might then be led to question their particular capacities in particular contexts or at particular times. For instance, where we think it is, was, or could be impaired. It may be obvious to a carer or a family member that the person they know with Alzheimer’s disease is quite lucid most mornings, and they might know this from many months or even years of experience. Perhaps the person’s capacity to understand or communicate deteriorates as the day goes on. In this case the carer or family member could quite reasonably claim that the person with Alzheimer’s is very capable of reasons-based behaviour at some times of the day, yet may struggle at other times. This sense of capacity to act, in contrast with instances of inability to perform acts, can be seen as central in diagnostic and conceptual criteria for rationality (Johnston and Liddle 2007; Korsgaard 2012).

The person who apparently acts irrationally, or without apparent reason can still offer reasons for their behaviour. In lieu of their explanations, or if explanations cannot be offered (or matched with the ensuing behaviour), reasons can still be found or at least presented and examined. For instance, the person who suffers from a delusion of persecution may act as though they are being persecuted. We might want to say that such behaviour is unreasonable in terms of the reality as we see it, including a reality as verified by other details, facts, experience, and expertise, i.e. where we can establish with some confidence that there is no evidence of persecution. This is not, however to say that no reasoning can be attributed to the person who behaves or acts as though they face persecution. In fact, by entering imaginatively into the experiences of the person who suffers the delusion, we can sometimes quite successfully predict their behaviour. While this may not always be the case, and not all such behaviour is so clearly guided by apparent and predictable reasons, there are often enough reasons to make the gaps between reason and action manageable and even meaningful, especially for those who seek to communicate with, support and care for those who are suffering from or with delusion.

In a highly technologised psychiatric approach, however, strange reasoning might be correlated with brain pathology with reference to a neurotypical brain derived from masses of data, by AI means. This might set in motion a more linear diagnostic pathway than one of finding meaning. Rather than reconstructing strange reasoning, and interpreting strange reasons from a point of view, the very presence of strangeness coupled with anomalous neural activity could be taken as explanation enough. The strangeness might simply be seen as the obvious symptomatic effect of a core pathology constituted by aberrant neural activity. This is not how psychiatry works now, but in the convergence of machine learning, AI, neuroscience, and psychiatry this reductionism is clearly possible (Bzdok and Meyer-Lindenberg 2018; Farahany 2011; Insel and Quirion 2005). The question becomes whether self-reports of an apparent victim of a persecution delusion might be given as much weight when neural data contradict or challenge those reports. Confidence in data processing, computer modelling, and neural recordings might tempt more reductivist psychiatrists to think of the interpersonal encounter as peripheral to an objective neural story. This might constitute an instance of *the symptom collapsing into the referent*.

The possibility that evidence might be evaluated differently at different times may be exacerbated by trust in the apparent objectivity of neural based technologies. In one case, a patient might be seen as exhibiting symptoms of mood disorder. In another, the patient may be judged to have a neural abnormality that leads to their reporting disordered mood. This would be a significant difference, rooted in the degree to which a reductionist paradigm in diagnosis came naturally or not, was accepted or not, by a practitioner. Both interpersonal and neural-based evaluative frameworks have something to contribute to a diagnostic situation, but the practical problem of integration remains. Recalling Simon Cohn (2016) from above, the issue is not to simply acquiesce in the seeming objectivity of the technical system.

The account we offer here suggests that not only are there varieties of reasons for doing or not doing x, but also that there can be varieties of evaluations that might lead two individuals to come to different conclusions about both the reasoning and the actions that follow. We can also see that a person’s reasoning may challenge or defy the reasoning of others, but that understanding about unusual reasons may yet be found. Even when in my evaluation I find a reason to act, and you find one to refrain, there is yet scope for understanding and for shared purpose. It is a possibility that there are as many evaluative frameworks as there are individuals evaluating. This suggests that when we try to understand one another, we are as much looking at the reasoning of the other as we are at their conclusions. Even when such reasoning appears to defy reality, meanings can be found. It remains unclear whether an AI can be developed to undertake this multifaceted appraisal of reasons and reasoning.

## Conclusion

In the interpretation of reasons, we deal with people’s practical conceptions of themselves, alongside evaluation of behaviour. Psychiatry can be informed by technology so as to provide insights otherwise hard to gain, which can aid in clinical decision support, evidence gathering through data analysis, and broadly in processes of assessing patients (Iniesta et al. 2016). But the technology ought not, without detailed interpretive discussion and questioning, be thought of as providing proof somehow of disorder (Maddox et al. 2019). This is unlikely to be the intent of many psychiatric practitioners, but in a context of highly technologized psychiatry, and a growing drive toward neurotechnology, the context may promote drift.

Technological solutions to human problems are best when they are reversible and based on careful consideration. Careful consideration allows decision-making to be constrained by practical conceptions of oneself, and so be grounded in good evaluations of reasons. This includes recognition of human complexity, whether in reasoning, or in the rich phenomenological experiences we have of these reasons. Where reasoning itself appears to be in doubt, psychiatry can offer analysis of disorders or recommend treatments when necessary, but the aim should always be to include the agent, and to presume agency.
